# Intubation and mortality prediction in hospitalized COVID-19 patients using a combination of convolutional neural network-based scoring of chest radiographs and clinical data

**DOI:** 10.1259/bjro.20210062

**Published:** 2022-03-24

**Authors:** Aileen O'Shea, Matthew D Li, Nathaniel D Mercaldo, Patricia Balthazar, Avik Som, Tristan Yeung, Marc D Succi, Brent P Little, Jayashree Kalpathy-Cramer, Susanna I Lee

**Affiliations:** ^1^ Department of Radiology, Massachusetts General Hospital, Boston, MA, United States; ^2^ Department of Radiology and Athinoula A. Martinos Center for Biomedical Imaging, Massachusetts General Hospital, Boston, United States; ^3^ Department of Radiology, Massachusetts General Hospital, Harvard Medical School, Boston, MA, United States; ^4^ Harvard Medical School, Boston, MA, United States; ^5^ Athinoula A. Martinos Center for Biomedical Imaging, MGH and BWH Center for Clinical Data Science, Department of Radiology, Harvard Medical School, Boston, MA, United States

## Abstract

**Objective:**

To predict short-term outcomes in hospitalized COVID-19 patients using a model incorporating clinical variables with automated convolutional neural network (CNN) chest radiograph analysis.

**Methods:**

A retrospective single center study was performed on patients consecutively admitted with COVID-19 between March 14 and April 21 2020. Demographic, clinical and laboratory data were collected, and automated CNN scoring of the admission chest radiograph was performed. The two outcomes of disease progression were intubation or death within 7 days and death within 14 days following admission. Multiple imputation was performed for missing predictor variables and, for each imputed data set, a penalized logistic regression model was constructed to identify predictors and their functional relationship to each outcome. Cross-validated area under the characteristic (AUC) curves were estimated to quantify the discriminative ability of each model.

**Results:**

801 patients (median age 59; interquartile range 46–73 years, 469 men) were evaluated. 36 patients were deceased and 207 were intubated at 7 days and 65 were deceased at 14 days. Cross-validated AUC values for predictive models were 0.82 (95% CI, 0.79–0.86) for death or intubation within 7 days and 0.82 (0.78–0.87) for death within 14 days. Automated CNN chest radiograph score was an important variable in predicting both outcomes.

**Conclusion:**

Automated CNN chest radiograph analysis, in combination with clinical variables, predicts short-term intubation and death in patients hospitalized for COVID-19 infection. Chest radiograph scoring of more severe disease was associated with a greater probability of adverse short-term outcome.

**Advances in knowledge:**

Model-based predictions of intubation and death in COVID-19 can be performed with high discriminative performance using admission clinical data and convolutional neural network-based scoring of chest radiograph severity.

## Introduction

Widespread vaccination for COVID-19 is underway; yet despite this, healthcare systems throughout many parts of the world continued to be overwhelmed by an escalating caseload.^1–5^ The upward trajectory of COVID-19 cases places an inexorable strain on hospital resources and is likely to continue to do so in the future with each surge cycle of the pandemic and viral variants. In caring for patients hospitalized for severe COVID-19 infection, clinical risk prediction tools to identify those most likely to decompensate in the short term would aid in optimizing the allocation of limited resources and minimize morbidity and mortality associated with the disease.

Numerous clinical prediction tools have been developed in a bid to better manage COVID-19 infection. Varying permutations have been modeled; but most use a combination of readily available clinical parameters, including laboratory tests (*e.g.,* white blood cell count, D-dimer, platelets) and demographic (*e.g.,* age) and medical history data (*e.g.,* vital signs, and comorbidities) to identify patients with symptomatic COVID-19 who are most at risk for decompensation.^6–8^ While most of these models have not been validated for generalizability, they have identified some common features as associated with the disease course including age, pulmonary, and cardiovascular status.^6,7^

Given that pulmonary infection is a hallmark of the illness, chest imaging has also been shown to correlate with outcomes. Models developed using chest CT assessment of disease burden along with clinical variables are reported as reasonably accurate, with AUC estimates greater than 0.8^9,10^; but, in most practice settings, chest CT is obtained only in a small subset of COVID-19 patients, usually as a secondary assessment in cases of negative RT-PCR results but persistent clinical suspicion of SARS-CoV-2 infection. Even when patients are symptomatic and hospitalized with severe disease, CT imaging is typically performed to assess associated complications, such as a pulmonary embolism, associated secondary infections, or sequela of barotrauma.^11^

In contrast, chest radiographs, obtained routinely when patients suspected of or known to be infected with COVID-19 present with symptoms, when used for prediction yield a model applicable to most hospitalized COVID-19 patients. Modeling using chest radiograph data in combination with clinical variables has been reported but their performance overall is less robust than chest CT.^12–18^ While there are many possibilities for this difference, one reason may be that chest radiograph assessment of disease severity is subject to greater observer variability. However, a convolutional neural network (CNN)-based algorithm that calculates a COVID-19 severity score for a chest radiograph based on the density and extent of lung opacities has been developed, is publicly available, and has been shown to correlate with disease severity assessment by multiple radiologists.^19–21^ Such an automated tool, *de novo* not subjective and, therefore, more reproducible than human readers, better lends itself to a model for outcome prediction that can eventually be scaled and tested in large cohorts in various clinical settings.

For the purpose of the study, we defined two different outcomes relative to hospital admission – death or intubation within 7 days and death within 14 days. Death and intubation are readily available and objective endpoints that indicate a severe disease course in patients with COVID-19. For the short-term outcome of 7 days, as some patients decompensated so quickly that they died before intubation, the two states were combined to describe a single outcome of critical COVID-19 illness. Such a marker indicating high likelihood of rapid decline would enable management planning, such as triage to more intensive monitoring and possibly prophylactic therapy. An assessment of likelihood of death within 14 days encompasses the entirety of the two-week period over which most COVD-19 positive patients presenting with symptoms decompensate into severe disease. Thus, it is a useful marker for healthcare resource allocation.

A CNN algorithm, for automated chest radiograph analysis, has been developed and validated as a surrogate for radiologist assessment and has been previously reported.^19–21^ The algorithm automates the chest radiograph interpretation yielding a reproducible and numerical output of the imaging information. With this tool on hand, we set out to identify whether demographic, clinical, and laboratory variables, in combination with a chest radiograph severity score from the CNN algorithm, could be used to predict outcomes that could be used to guide management of hospitalized COVID-19 positive patients.

## Methods and materials

### Cohort definition and follow-up

Institutional review board (IRB) approval was obtained and the requirement for informed consent waived for this HIPAA compliant study. We performed a retrospective analysis of consecutive adult patients (age≥18 years old) admitted to our hospital system between 14 March 2020 and 21 April 2020 who were diagnosed with COVID-19 by the reverse transcription-polymerase chain reaction assay before the time of or within four days following admission. Patients either presented through the emergency department or were transferred from outside our hospital system. All patients were followed to date of discharge or death. Patients who were alive and not discharged were followed until 15 September 2020.

### Data collection

We queried our institution clinical data repository to extract the following demographic, clinical, and laboratory variables: age, gender, temperature, body mass index (BMI), oxygen saturation, ICU admission date, death date, comorbidities, ventilation status, fibrinogen, estimated glomerular filtration rate (eGFR), lactate dehydrogenase (LDH), platelets (PLT), prothrombin time (PT), white blood cells (WBC), D-dimer and C-reactive protein (CRP).

We used International Classification of Diseases, Tenth Revision codes (ICD-10) to extract comorbidities (Supplementary Material 1), including diabetes, cancer, hypertension, cardiac disease, and respiratory disease (chronic obstructive pulmonary disease or emphysema and asthma). Laboratory values recorded were those closest to the admission date. Extracted laboratory values were those available within 7 days of admission; however, only the value closest to the date of admission was included. All Patients Refined Diagnosis-Related Groups (APRDRG) and ICD-10 codes were used to extract ventilation status (Supplementary Material 1). These variables were cross-referenced with thorough manual review of the electronic health record (EHR).

### Outcome measures

Two outcome variables of short-term disease progression were defined: death or intubation within 7 days of admission and death within 14 days of admission.

### Chest radiograph scoring

**Figure 1.**
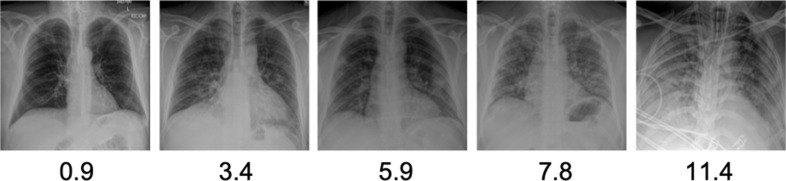
Chest radiograph with their corresponding severity scores from the CNN algorithm. The automated severity score uses a Siamese CNN by using raw DICOM pixel data to calculate a numeric score for lung disease. An increasing score (left to right) corresponds to increasing parenchymal lung opacity and extent. The score is continuous; however, a severity scale proposed from radiologist interpretation is as follows: ≤2.5 no or minimal disease, >2.5 and ≤5.0 mild disease, >5.0 and ≤9.0 moderate disease, and>9.0 severe disease.^19–21^

Chest radiographs included in this analysis were those available either up to 2 days prior to or 5 days post admission. If more than one exam was available, the chronologically earliest radiograph within this interval was analyzed. Chest radiographs with an endotracheal tube in place were excluded from modeling for intubation. An automated severity score was generated for each of the chest radiographs determined from the density and extent of lung opacities using a the CNN algorithm, previously validated in multiple patient populations using the manual assessments of disease severity by multiple radiologists as a reference standard.^19^ This algorithm receives raw DICOM pixel data from frontal chest radiographs as inputs and calculates a numeric score for lung disease severity. While the score is continuous, as a guide for interpretation, the following ranges of scores reflect different gradations of severity as established by our radiologists: ≤2.5 no or minimal disease, >2.5 and ≤5.0 mild disease, >5.0 and ≤9.0 moderate disease, and >9.0 severe disease. Examples of chest radiograph scores for representative images are shown in Figure 1.

### Statistical analysis

Descriptive summaries were generated for continuous and categorical variables; continuous variables were summarized as median and IQR (interquartile range) and categorical variables as frequencies (percentages). The percentage of missing data were also calculated for each variable.

Missing data were present in at least of the one candidate predictors in 23% of the study population. Multiple imputation (MI) was used to account for the missing exposure values. Missing exposure data were “filled-in” using observed exposure data via a chained equation approach.^22^ To account for the uncertainty in the exposure data, this imputation process was repeated 100 times resulting in the creation of 100 complete data sets. Outcome data were excluded from the imputation process.

For each imputed data set, a penalized (LASSO) logistic regression model was constructed. The model was chosen over a standard logistic model because of its ability to shrink parameter estimates that are not, or at most weakly, associated with the outcome of interest to zero. Thus, this approach simultaneously performs variable estimation and variable selection (*e.g.,* variables whose estimates are zero are effectively dropped from the model). 10-fold cross-validation was performed to estimate all model parameters (both covariate effects and the tuning parameter). Parameter estimates and cross-validated model predictions were stored for each imputed data set. Additional details about the missing data patterns, the analysis approach (*e.g.,* tuning parameter selection, cross-validation), comparator models (linear *vs* non-linear coding of continuous variables, main-effects only *vs* interactions), and estimation methods (LASSO, random forests) are provided in the supplemental documentation.

**Figure 2.**
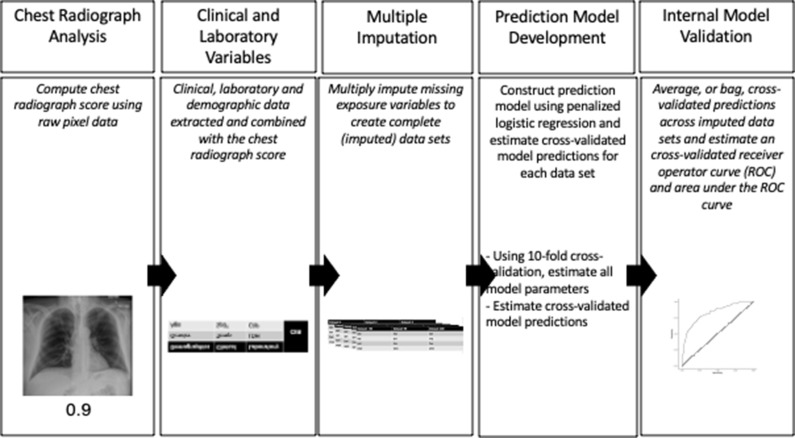
Data collection and analysis. A numeric score of the severity of COVID-19 on each chest radiograph was obtained using the convolutional neural network (CNN) algorithm. Demographic, clinical, laboratory, and chest radiograph score were combined to impute 100 datasets. Penalized logistic regression modeling was performed on each dataset to identify relevant predictors of each outcome. 10-fold cross-validation was performed to identify all model parameters and to estimate model predictions. Cross-validated predictions were averaged, or bagged, across datasets to estimate cross-validated ROC curves and AUC estimates.

Parameter estimates and cross-validated predictions were averaged, or bagged, across imputed data sets. The importance of each variable was assessed by ranking the absolute value of the average estimates as well as estimating the percentage of times a non-zero estimate was obtained. Average predictions, and the true outcome status, were then used to estimate the cross- validated AUC curves and their 95% confidence intervals. A schematic diagram of the data collection process and data analysis is presented in Figure 2.

Cross-fold validation rather than splitting our dataset was chosen for internal validation as this represents the current standard regardless of sample size. The former method avoids the possibility that resultant validation metric (AUC, RMSE, etc) may be biased simply due to the choice of the test set.

The study cohort includes 315 patients (39%) whose chest radiographs were used to develop the CNN algorithm.^19^ To quantify the possible effect of data leakage (or double dipping), the proposed analyses was performed on both the full cohort and on the subgroup of patients whose chest radiographs were not used in developing the CNN algorithm.

All analyses were performed using R 4.0.2 (R Core Team (2020). R: A language and environment for statistical computing. R Foundation for Statistical Computing, Vienna, Austria. URL https://www.R-project.org/) and the mice, glmnet, pROC, randomForest, and caret libraries.^22–27^

## Results

### Cohort description

Table 1 describes the diagnostic variables for the entire cohort. 801 patients (median age 59, interquartile range 46–73 years; 469 men). The median BMI for admitted patients was 28.9 (IQR, 25.1–33.6) which implies many of these patients were classified as either overweight or obese. Cardiac disease, hypertension and diabetes were the most frequently encountered comorbidities, present in 62%, 59%, and 38% of patients, respectively. The admission chest radiograph was on the day of hospitalization for 691 patients (86%) and within 1 day for 722 (90%) of the cohort. The median score for the admission chest radiograph was 4.21 (IQR 2.21–6.81), corresponding to mild disease. Fibrinogen, prothrombin time and D-dimer values were available within 2 days of admission for 43%, 68%, and 89% of the cohort, respectively. GFR, LDH, PLT, WBC and CRP values were available within two days of admission for 98%, 95%, 99%, and 95% of the cohort, respectively.

**Table 1.** Diagnostic variables collected from the cohort at admission. Continuous variables are summarized by median and IQR (interquartile range) and categorical variables as frequencies (proportions). The percentage (%) of missing data for each variable collected is provided

	Variables	Overalla	Missing (%)b
N		801	
Demographics	Age	59 [46, 73]	0
	Gender (Male)	469 (58.6)	0
	BMI	28.9 [25.1, 33.6]	7.2
Comorbidities	COPD	83 (10.4)	0
	Asthma	107 (13.4)	0
	Emphysema	26 (3.2)	0
	Hypertension	476 (59.4)	0
	Cancer	109 (13.6)	0
	Diabetes	305 (38.1)	0
	Cardiac disease	497 (62.0)	0
CXR Score	Admission	4.21 [2.21, 6.81]	8.1c
Labs	WBC	6.52 [5.09, 8.49]	0.9
	D- Dimer	974 [602, 1631]	5.5
	CRP	74.3 [34.2, 144.3]	5.7
	LDH	326 [246, 436]	3.6
	Platelets	199 [157, 255]	1
	Estimated GFR	82 [57, 100]	1.2
Symptoms	SpO_2_ (%)	95 [93, 97]	1.1
	Temperature (°C)	36.9 [36.4, 37.4]	3.7

a Continuous variables are summarized by median and IQR (interquartile range) and categorical variables as frequencies (proportions).

b The percentage (%) of missing data for each variable collected is provided.

c In these patient’s admission chest radiograph demonstrated an endotracheal tube *in situ*, which was an exclusionary criterion.

### Intubation or death within 7 days

A total of 243 (30.3%) patients were either intubated or died by day 7. Of these, 36 patients (4.5%) died and 207 (25.8%) had been intubated (Table 2). Table 3 summarizes the discriminative performance of the prediction models. The cross-validated AUCs were approximately 0.80 for all evaluated models and estimation methods. Given the similarity of these results, specific details pertaining to the penalized logistic regression model utilizing only main effects (no interactions) and continuous variables modeled as linear terms are summarized.

**Table 2.** Vital status at 7- and 14-day post admission and intubation status at 7 days post admission for the cohort

Outcomes (*N* = 801)	Value	Frequency (%)
**Vital status / intubated at 7 days post admission**	Alive and not intubated	558 (69.7)
	Deceased and/or intubated	243 (30.3)
	Intubated and alive at 7 days	207 (25.8)
	Deceased at 7 days without intubation	25 (3.1)
	Intubated and deceased at 7 days	11 (1.4)
**Vital status 14 days post admission**	Alive	736 (91.9)
	Deceased	65 (8.1)

**Table 3.** Cross-fold validated AUC estimates (95% confidence intervals) by model parameterization (*e.g.,* continuous variables modeled as linear or non-linear terms, and all two-way interactions or no interactions between predictors) and estimation method

		Intubation or death within 7 days	Death within 14 days
Penalized (LASSO) Logistic Regression	Linear: No Interactions	0.82 (0.79, 0.86)	0.82 (0.78, 0.87)
	Non-linear: No Interactions	0.82 (0.79, 0.85)	0.82 (0.77, 0.86)
	Linear: All 2-way Interactions	0.81 (0.78, 0.84)	0.79 (0.74, 0.83)
Random Forests		0.81 (0.78,0.84)	0.83 (0.79, 0.87)

The average standardized regression coefficients of this model are presented in Table 4. Chest radiograph severity score was positively associated with death or intubation within 7 days. Clinical variables that also demonstrated a positive association included the presence of cardiovascular disease, hypertension, and diabetes. Laboratory values that demonstrated a positive association included CRP, LDH, WBC count, and D-dimer whereas eGFR, SpO_2_ (oxygen saturation) and platelets demonstrated a negative association, indicating a protective effect of a higher value.

**Table 4.** Average standardized penalized regression coefficients by variable category and outcome (LASSO: linear, no interaction model)

		Intubation or death within 7 **days**		Death within 14 **days**	
**Category**	**Variable**	Inclusion* (%)	**Average Standardized Estimate**	**Inclusion (%)**	**Average Standardized Estimate**
	(Intercept)	100	−1.035	100	−2.741
Demographics	Age	0	0.000	100	0.581
	Gender	0	0.000	0	0.000
	BMI	2	0.000	0	0.000
Comorbidities	Diabetes	100	0.067	0	0.000
	Cancer	0	0.000	0	0.000
	Hypertension	95	0.015	0	0.000
	Cardiac Disease	100	0.610	0	0.000
	COPD	0	0.000	20	0.001
	Emphysema	0	0.000	0	0.000
	Asthma	0	0.000	0	0.000
Chest X-ray (CXR) Score	First score within [−2,5] days of hospital admission	100	0.4346	84	0.054
Labs					
	eGFR	100	−0.106	100	−0.405
	LDH	100	0.619	0	0.000
	Platelets	98	−0.050	0	0.000
	WBC	100	0.107	1	0.000
	D-Dimer	3	0.000	0	0.000
	CRP	100	0.402	0	0.000
Symptoms	SpO_2_	100	−0.184	79	−0.028
	Temperature (°C)	80	0.026	0	0.000

Footnote: *Inclusion denotes the percentage of times the variable had a non-zero parameter estimate across 100 imputed data sets.

**Figure 3.**
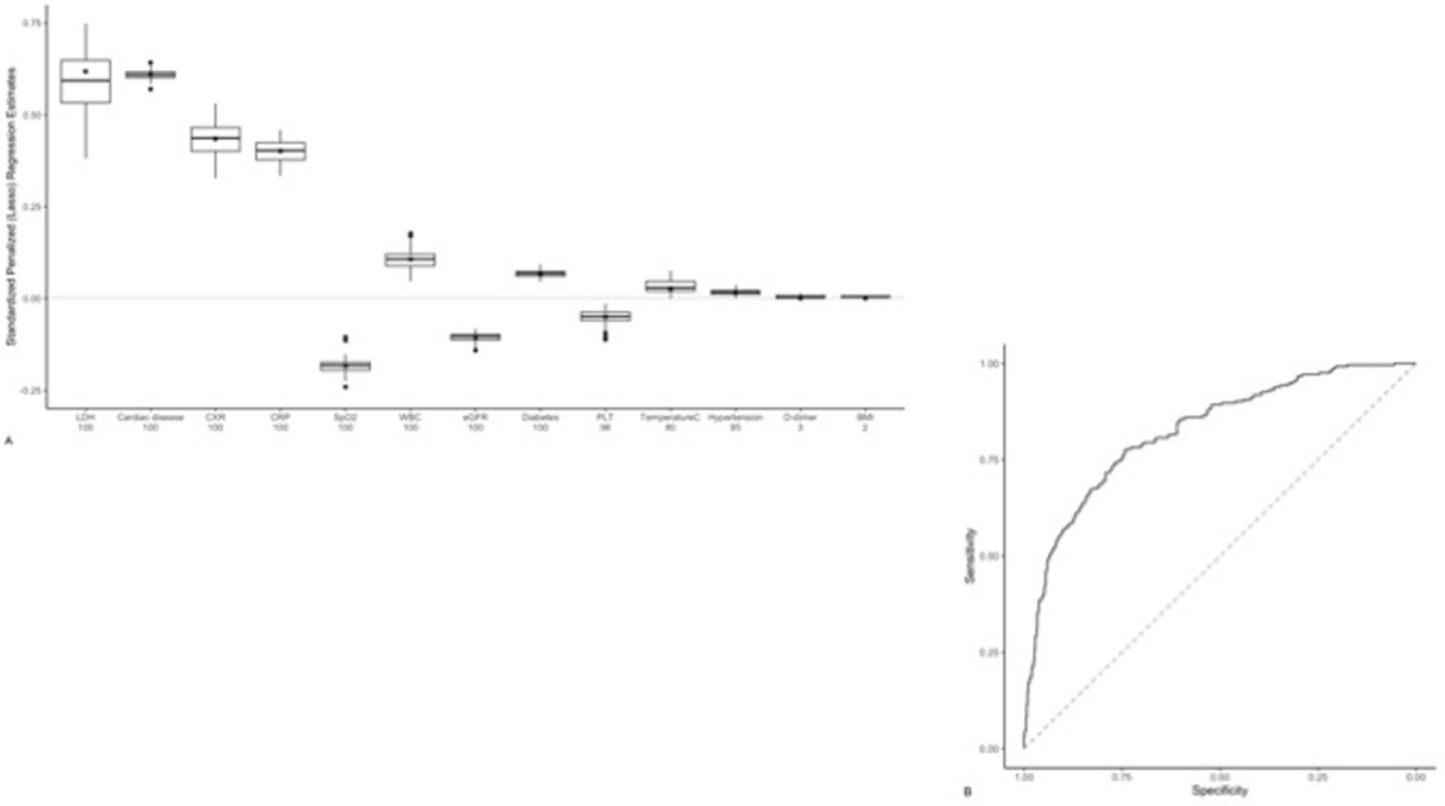
A. Model predicting intubation or death within 7 days. Distribution of penalized logistic regression estimates across 100 imputed data sets are shown. Average standardized estimates (*e.g.,* higher LDH, CRP or CXR score and lower SpO_2_, eGFR, platelets (PLT) values) were associated with an increased odds of being intubated or death within 7 days. LDH, cardiac disease, CXR, CRP, SpO_2_, WBC, and eGFR values were deemed important, as defined as both the absolute value of the standardized regression coefficient and the percentage of times the estimate was non-zero (reported in the X-axis below each variable). The asterisk corresponds to the average estimate, including those reduced to zero via the LASSO algorithm (Table 4). (**B.**) ROC curve of intubation or death within 7 days of hospital admission using bagged cross-validated predictions and the true outcome status (AUC: 0.82 [95% CI: 0.79–0.86], Table 3).

Figure 3A illustrates the distribution of average standardized regression coefficients for each variable and the percentage of times, across the imputed data sets, each variable had a non-zero estimate. In defining the importance of a predictor both in terms of the absolute value and the percentage of times a non-zero estimate was seen, cardiac disease, CXR and CRP, SpO_2_, WBC, and eGFR were deemed important predictors of death or intubation within 7 days of hospital admission. The cross-validated ROC curve for this model is presented in Figure 3B and its associated AUC is 0.82 (95% CI, 0.79–0.86) (Table 3).

### Death within 14 days

Sixty-five (8.1%) patients died by day 14 (Table 2). Chest radiograph severity score was positively associated with this outcome (Table 4). The only clinical variable that also demonstrated a positive association was age. eGFR and SpO_2_ values showed a negative association, thereby indicating a protective effect of a higher value.

**Figure 4.**
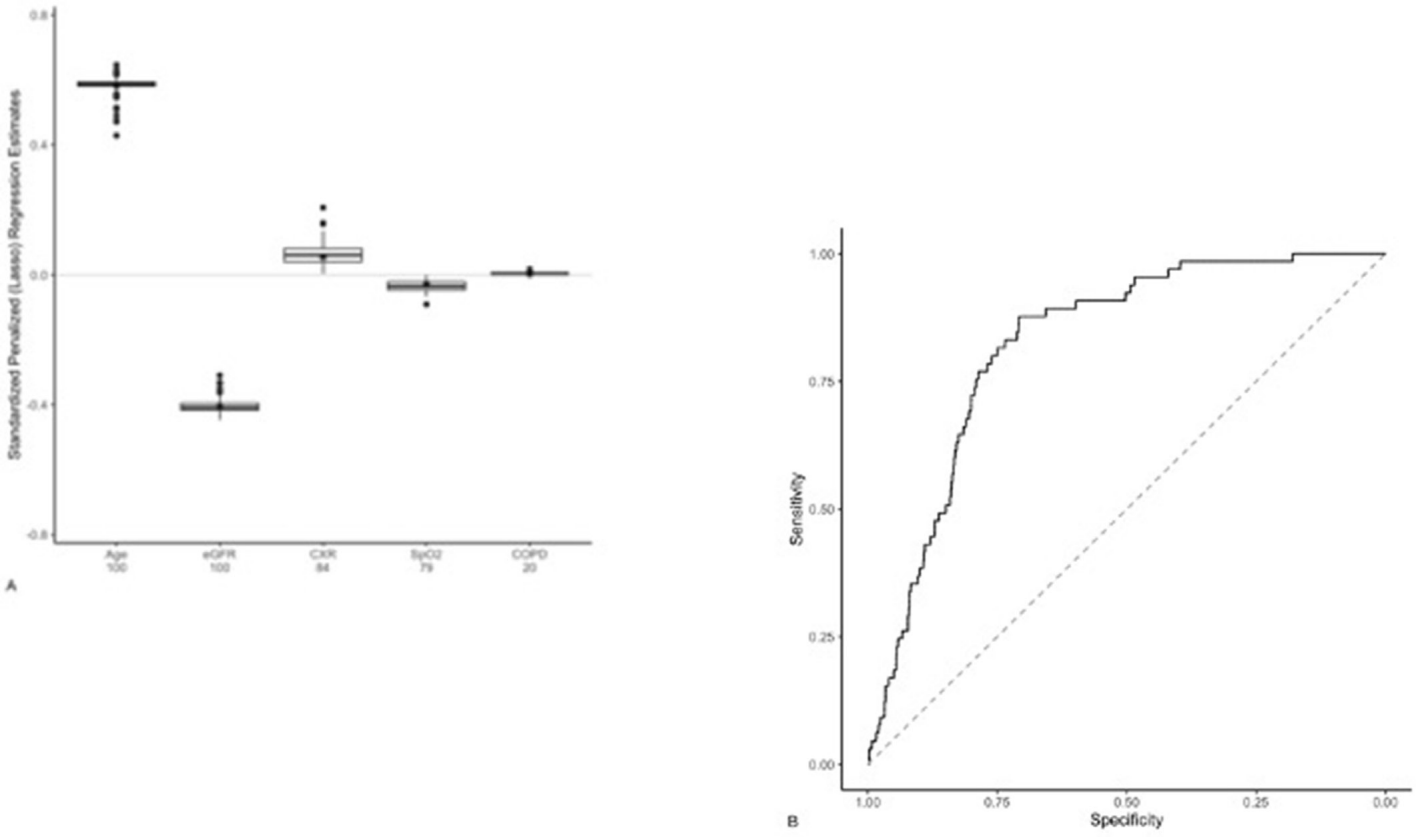
A. Model predicting death within 14 days. Distribution of penalized logistic regression estimates across 100 imputed data sets are shown. Average standardized estimates (*e.g.,* increased age and lower eGFR values) were associated with death within 14 days. Age, eGFR CXR and SpO_2_ values deemed important, as defined as both the absolute value of the standardized regression coefficient and the percentage of times the estimate was non-zero (reported in the X-axis below each variable). The asterisk corresponds to the average estimate, including those reduced to zero via the LASSO algorithm (Table 4). (**B.**) ROC curve of death within 14 days of hospital admission using bagged cross-validated predictions and the true outcome status (AUC: 0.82 [95% CI: 0.78–0.87], Table 3).

Age, eGFR, CXR, and SpO_2_ values were all deemed important predictors of death within 14 days of hospital admission (Figure 4A). The cross-validated ROC curve for this model is presented in Figure 4B and its associated AUC is 0.82 (95% CI, 0.78–0.87) (Table 3).

### Subgroup analysis

Table 5 summarizes the cross-validated AUC estimates by estimation method (LASSO, random forests) and by model complexity for the subgroup of 486 patients not included in the CNN algorithm development. We assume that the results of the random forests are the surrogates for the gold-standard since this estimation approach does not require the explicit modeling of covariate effects, or their interactions. For intubation or death with 7 days, the cross-validated AUCs for the full and subgroup cohorts were 0.81 (95% CI, 0.78–0.84) and 0.80 (95% CI, 0.76–0.85), respectively. Similarly, for death within 14 days, the cross-validated AUCs for the full and subgroup cohorts were 0.83 (95% CI, 0.79–0.87) and 0.83 (95% CI, 0.78–0.88), respectively. Due to the similarity of these estimates, the data leakage was adequately accounted for in the analysis approach (*i.e.,* via the use of cross-fold validation). Additional summaries for this subgroup are presented in the Supplementary Materials.

**Table 5.** Cross-fold validated AUC estimates (95% confidence intervals) by model parameterization (*e.g.,* continuous variables modeled as linear or non-linear terms, and all two-way interactions or no interactions between predictors) and estimation method for the subgroup of patients not included in the CNN algorithm development

		Intubation or death within 7 days	Death within 14 days
Penalized (LASSO) Logistic Regression	Linear: No Interactions	0.81 (0.76, 0.85)	0.77 (0.71, 0.83)
	Non-linear: No Interactions	0.80 (0.75, 0.84)	0.79 (0.72, 0.85)
	Linear: 2-way Interactions	0.78 (0.73, 0.82)	0.78 (0.72, 0.84)
Random Forests		0.80 (0.76, 0.85)	0.83 (0.78, 0.88)

## Discussion

Our study showed that in patients hospitalized for COVID-19, readily available data obtained early in admission (*i.e.,* demographics, major co-morbidities, vital signs, laboratory values, and a severity score of the chest radiograph generated by a CNN-based algorithm) can predict the likelihood of decompensation to severe illness. Models of intubation or death within 7 days and of death within 14 days both showed a cross-validated discriminative performance of 0.82. As stated above, there was a cohort overlap of 315 patients. In order to address concerns regarding data leakage, analyses were performed in the full cohort, and in a subgroup excluding overlapping subjects. In each model, including modeling of the subgroup not used to develop the CNN algorithm, the automated chest radiograph severity score was identified as an important predictor that was positively associated with patient outcome. Oxygen saturation and eGFR were also seen as important predictors for both outcomes.

Previous studies on the usefulness of a chest radiograph in prognosticating early progression of COVID-19 to critical illness have produced varying results. A UK-based study that examined the chest radiographs of over 1000 patients admitted to a tertiary academic hospital in mid-March to mid-April 2020 failed to demonstrate any significant or clinically meaningful association between chest radiograph findings and 30-day outcomes of death or hospital discharge.^28^ Scoring systems for chest radiographs elsewhere have been successfully used semi-quantitative scoring tools in order to predict the likelihood of admission to hospital and/or death.^17^ Typically this is performed by dividing the chest radiograph into separate zones and scoring each section. For young patients (aged 21–50 years), a higher chest radiograph score (>3) was positively associated with hospital admission.^17^ While in another study, a higher chest radiograph score was positively associated with death.^29^ However, in both studies, the involved a small number of radiologists in a single institution.^17,29^

“In contradistinction to reader-based scoring systems, AI based scoring of chest radiographs offer the advantages of greater reproducibility, and scalability. At the outset of the pandemic, the diagnostic performance of AI based systems as compared to reader-based chest radiograph scoring systems has been evaluated elsewhere and was shown to be independent and comparable predictors of adverse outcomes in patients with COVID-19.^30^ More recent studies have built on this initial experience to develop AI- based radiograph analysis that out-performs, radiologist derived scores and clinical variables.^31,32^ A notable shortcoming of the study by Jiao and colleagues, was the dichotomization of outcomes into critical and non-critical. In contrast, our study aimed to assist in the stratification of all hospitalized patients with COVID-19, to assist in the allocation of critical care resources by identifying those at most risk for decompensation. When integrated with clinical or laboratory values, predictive models with modest discriminative performance for hospitalization outcomes including critical illness and death, have been reported with AUC’s of 0.66 and 0.59, respectively, reported elsewhere.^15^ Our model adds to the growing evidence that AI scoring of chest radiographs are an important variable in models that assess COVID-19 severity early in the course of illness.”

Models using reader-based scoring of chest radiographs, even when successful, cannot be easily generalized. Scaling such models for validation in larger cohorts and in other clinical settings would be challenging given the manual and subjective nature of the chest radiograph input. If validated, implementation into a clinical workflow would involve chest radiograph severity scores from numerous radiologists introducing observer variability as a factor in the model’s performance. In contrast, our models depend upon a chest radiograph severity score automatically derived from a CNN-based algorithm that analyzes the DICOM inputs of the radiographic dataset. Thus, the imaging output becomes objective, reproducible, and scalable and is now amenable to high-throughput dissemination, similar to laboratory testing. The use of a CNN to generate a numeric and continuous variable also provides an opportunity to dynamically model outcomes as the clinical profile of patients with severe COVID-19 evolves with viral mutations, public immunization, and novel therapies.^33^

With respect to other prediction models that have investigated the association between critical illness and clinical and/or laboratory values, our results are mostly congruent. Established risk factors for critical illness in larger cohorts from the UK and US suggest a similar positive association between demographic variables (age, BMI, and co-morbidities), laboratory variables (CRP, D-dimer), and clinical variables such as admission oxygen saturations.^34–36^ The importance of eGFR in modeling outcomes has not been previously noted but has not been included in many previous modeling studies. It may serve as a surrogate marker of cardiovascular disease, hypertension, or diabetes, and as a continuous variable that can be readily and objectively verified, may be a more informative predictor than the clinical history.

Our study has several important limitations. Most important is the single center design and absence of an external test set. Indeed, these concerns have been highlighted extensively in more recent reviews examining the available body of evidence for deep-learning-based assessment of COVID-19 chest radiographs, noting that single-center bias, differences in technical parameters and the presence of chest radiograph artifacts, which may hamper the reliability of many available deep learning models.^37^ Moreover, the data were collected from patients presenting early in the pandemic when COVID management and prognosis was quite different, at least as it has evolved within our practice setting. These considerations limit the generalizability of our prediction model. However, given the heterogeneity of the COVID-19 surges and medical practice during the duration of this pandemic, obtaining sufficiently complete and homogeneous datasets with outcome data to validate generalizability has not yet been feasible.^38^ The CNN algorithm, however, has been validated previously in separate populations.^21^ While clinical and demographic variables were retrieved in the majority of patients, these values were missing in some. We assumed the missing data mechanism was at random and accounted for variable missingness using multiple imputation. If this assumption is not satisfied, then additional data would need to be collected to properly handle the missing data. We did not gather patient symptoms at presentation as they were variably recorded. Thus, it is possible that patients without COVID-19-related symptoms hospitalized for other conditions could have been included in our cohort. However, our institution was undergoing a COVID-19 surge during the accrual period. Patients requiring non-COVID related hospitalizations were being diverted, if possible, to other centers, non-emergent procedures requiring hospitalization had been canceled, and those testing positive for COVID-19 but without symptoms were being monitored as outpatients virtually. Thus, nearly all of the patients with COVID-19 positive tests hospitalized during this period were admitted for this diagnosis. Finally, other factors previously noted as important predictors, such as duration of symptoms prior to presentation and chest CT findings, were not included in our modeling as the data were not available on most of the cohort.

Our models identify COVID-19 patients at risk of progressing to intubation or death within the first two weeks of hospitalization. Predictors are clinical, laboratory, and radiographic data routinely obtained at the point of admission. The chest radiograph scoring of disease severity is an important predictor and, as it is automated and CNN-based, is numerical and readily scaled into a high throughput clinical workflow, similar to the other laboratory values. If validated, the model could be used to help inform resource allocation and clinical practice algorithms in settings where a surge in case burden strains hospital resources.

## References

[1] Looi MK . Covid-19: Is a second wave hitting Europe BMJ 2020; 371: m4113. doi: 10.1136/bmj.m4113 33115704

[2] Argulian E . Anticipating the "Second Wave" of Health Care Strain in the COVID-19 Pandemic. JACC Case Rep 2020; 2: 845–46. doi: 10.1016/j.jaccas.2020.04.005 32296782 PMC7158795

[3] Ali I . COVID-19: Are We Ready for the Second Wave Disaster Med Public Health Prep 2020; 14: e16–18. doi: 10.1017/dmp.2020.149 32379015 PMC7239772

[4] Mulligan MJ, Lyke KE, Kitchin N, Absalon J, Gurtman A, Lockhart S, et al . Phase I/II study of COVID-19 RNA vaccine BNT162b1 in adults. Nature 2020; 586: 589–93. doi: 10.1038/s41586-020-2639-4 32785213

[5] . Jackson LA, Anderson EJ, Rouphael NG, Roberts PC, Makhene M, Coler RN, et al . An mRNA Vaccine against SARS-CoV-2 - Preliminary Report. N Engl J Med. 2020;383(20):1920-31. 32663912 10.1056/NEJMoa2022483 PMC7377258

[6] Clift AK, Coupland CAC, Keogh RH, Diaz-Ordaz K, Williamson E, Harrison EM, et al . Living risk prediction algorithm (QCOVID) for risk of hospital admission and mortality from coronavirus 19 in adults: national derivation and validation cohort study. BMJ 2020; 371: m3731. doi: 10.1136/bmj.m3731 33082154 PMC7574532

[7] Knight SR, Ho A, Pius R, Buchan I, Carson G, Drake TM, et al . Risk stratification of patients admitted to hospital with covid-19 using the ISARIC WHO Clinical Characterisation Protocol: development and validation of the 4C Mortality Score. BMJ 2020; 370: m3339. doi: 10.1136/bmj.m3339 32907855 PMC7116472

[8] Wynants L, Van Calster B, Collins GS, Riley RD, Heinze G, Schuit E, et al . Prediction models for diagnosis and prognosis of covid-19: systematic review and critical appraisal. BMJ 2020; 369: m1328. doi: 10.1136/bmj.m1328 32265220 PMC7222643

[9] Colombi D, Bodini FC, Petrini M, Maffi G, Morelli N, Milanese G, et al . Well-aerated Lung on Admitting Chest CT to Predict Adverse Outcome in COVID-19 Pneumonia. Radiology 2020; 296: E86–96. doi: 10.1148/radiol.2020201433 32301647 PMC7233411

[10] Grodecki K, Lin A, Cadet S, McElhinney PA, Razipour A, Chan C, et al . Quantitative Burden of COVID-19 Pneumonia on Chest CT Predicts Adverse Outcomes: A Post-Hoc Analysis of a Prospective International Registry. Radiol Cardiothorac Imaging 2020; 2: e200389. doi: 10.1148/ryct.2020200389 33778629 PMC7605078

[11] American College of Radiology [Available from. Available from: https://www.acr.org/Advocacy-and-Economics/ACR-Position-Statements/Recommendations-for-Chest-Radiography-and-CT-for-Suspected-COVID19-Infection

[12] Balbi M, Caroli A, Corsi A, Milanese G, Surace A, Di Marco F, et al . Chest X-ray for predicting mortality and the need for ventilatory support in COVID-19 patients presenting to the emergency department. Eur Radiol 2021; 31: 1999–2012. doi: 10.1007/s00330-020-07270-1 33033861 PMC7543667

[13] Schalekamp S, Huisman M, van Dijk RA, Boomsma MF, Freire Jorge PJ, de Boer WS, et al . Model-based Prediction of Critical Illness in Hospitalized Patients with COVID-19. Radiology 2021; 298: E46–54. doi: 10.1148/radiol.2020202723 32787701 PMC7427120

[14] Al-Smadi AS, Bhatnagar A, Ali R, Lewis N, Johnson S . Correlation of chest radiography findings with the severity and progression of COVID-19 pneumonia. Clin Imaging 2021; 71: 17–23: S0899-7071(20)30432-0. doi: 10.1016/j.clinimag.2020.11.004 33166898 PMC7644185

[15] Kwon YJF, Toussie D, Finkelstein M, Cedillo MA, Maron SZ, Manna S, et al . Combining Initial Radiographs and Clinical Variables Improves Deep Learning Prognostication in Patients with COVID-19 from the Emergency Department. Radiol Artif Intell 2021; 3: e200098. doi: 10.1148/ryai.2020200098 33928257 PMC7754832

[16] Fontanellaz M, Ebner L, Huber A, Peters A, Löbelenz L, Hourscht C, et al . A Deep-Learning Diagnostic Support System for the Detection of COVID-19 Using Chest Radiographs: A Multireader Validation Study. Invest Radiol 2021; 56: 348–56. doi: 10.1097/RLI.0000000000000748 33259441

[17] Toussie D, Voutsinas N, Finkelstein M, Cedillo MA, Manna S, Maron SZ, et al . Clinical and Chest Radiography Features Determine Patient Outcomes in Young and Middle-aged Adults with COVID-19. Radiology 2020; 297: E197–206. doi: 10.1148/radiol.2020201754 32407255 PMC7507999

[18] Borakati A, Perera A, Johnson J, Sood T . Diagnostic accuracy of X-ray versus CT in COVID-19: a propensity-matched database study. BMJ Open 2020; 10(11): e042946. doi: 10.1136/bmjopen-2020-042946 PMC7650091 33158840

[19] Li MD, Arun NT, Gidwani M, Chang K, Deng F, Little BP, et al . Automated Assessment and Tracking of COVID-19 Pulmonary Disease Severity on Chest Radiographs using Convolutional Siamese Neural Networks. Radiol Artif Intell 2020; 2: e200079. doi: 10.1148/ryai.2020200079 33928256 PMC7392327

[20] Li MD, Arun NT, Gidwani M, Chang K, Deng F, Little BP, et al . Automated assessment of COVID-19 pulmonary disease severity on chest radiographs using convolutional Siamese neural networks. MedRxiv 2020: 2020.05.20.20108159. doi: 10.1101/2020.05.20.20108159 PMC7392327 33928256

[21] Li MD, Arun NT, Aggarwal M, Gupta S, Singh P, Little BP, et al . Improvement and Multi-Population Generalizability of a Deep Learning-Based Chest Radiograph Severity Score for COVID-19. medRxiv. MedRxiv 2020: 2020.09.15.20195453. doi: 10.1101/2020.09.15.20195453 PMC9302282 35866818

[22] van Buuren S . Flexible Imputation of Missing Data, Second Edition. Second edition. ed. Second edition. | Boca Raton, Florida : CRC Press, [2019] |: CRC Press, Taylor & Francis Group; 2018. doi: 10.1201/9780429492259

[23] Robin X, Turck N, Hainard A, Tiberti N, Lisacek F, Sanchez J-C, et al . pROC: an open-source package for R and S+ to analyze and compare ROC curves. BMC Bioinformatics 2011; 12(1): 77. doi: 10.1186/1471-2105-12-77 21414208 PMC3068975

[24] Friedman J, Hastie T, Tibshirani R . Regularization Paths for Generalized Linear Models via Coordinate Descent. J Stat Softw 2010; 33: 1–22. doi: 10.18637/jss.v033.i01 20808728 PMC2929880

[25] . Liaw A, Wiener M. Classification and regression by randomForest. R news. 2002;2(3):18-22.

[26] Kuhn M . Building Predictive Models in R Using the caret Package. J Stat Softw 2008; 28: 1–26. doi: 10.18637/jss.v028.i05 27774042

[27] mice G-O . Multivariate Imputation by Chained Equations in R. J Stat Softw 2011; 45: 1–67. doi: 10.18637/jss.v045.i03

[28] Borakati A, Perera A, Johnson J, Sood T . Chest X-Ray Has Poor Sensitivity and Prognostic Significance in COVID-19: A Propensity Matched Database Study. Emergency Medicine July 7, 2020; 2020: 2020. 10.1101/2020.07.07.20147934

[29] Maroldi R, Rondi P, Agazzi GM, Ravanelli M, Borghesi A, Farina D . Which role for chest x-ray score in predicting the outcome in COVID-19 pneumonia Eur Radiol 2021; 31: 4016–22. doi: 10.1007/s00330-020-07504-2 33263159 PMC7707903

[30] Mushtaq J, Pennella R, Lavalle S, Colarieti A, Steidler S, Martinenghi CMA, et al . Initial chest radiographs and artificial intelligence (AI) predict clinical outcomes in COVID-19 patients: analysis of 697 Italian patients. Eur Radiol 2021; 31: 1770–79. doi: 10.1007/s00330-020-07269-8 32945968 PMC7499014

[31] Jiao Z, Choi JW, Halsey K, Tran TML, Hsieh B, Wang D, et al . Prognostication of patients with COVID-19 using artificial intelligence based on chest x-rays and clinical data: a retrospective study. Lancet Digit Health 2021; 3: e286–94: S2589-7500(21)00039-X. doi: 10.1016/S2589-7500(21)00039-X 33773969 PMC7990487

[32] Tan T, Das B, Soni R, Fejes M, Yang H, Ranjan S, et al . Multi-modal trained artificial intelligence solution to triage chest X-ray for COVID-19 using pristine ground-truth, versus radiologists. Neurocomputing 2022; 485: 36–46. doi: 10.1016/j.neucom.2022.02.040 35185296 PMC8847079

[33] Pianykh OS, Langs G, Dewey M, Enzmann DR, Herold CJ, Schoenberg SO, et al . Continuous Learning AI in Radiology: Implementation Principles and Early Applications. Radiology 2020; 297: 6–14. doi: 10.1148/radiol.2020200038 32840473

[34] Petrilli CM, Jones SA, Yang J, Rajagopalan H, O’Donnell L, Chernyak Y, et al . Factors associated with hospital admission and critical illness among 5279 people with coronavirus disease 2019 in New York City: prospective cohort study. BMJ 2020; 369: m1966. doi: 10.1136/bmj.m1966 32444366 PMC7243801

[35] Seidu S, Gillies C, Zaccardi F, Kunutsor SK, Hartmann-Boyce J, Yates T, et al . The impact of obesity on severe disease and mortality in people with SARS-CoV-2: A systematic review and meta-analysis. Endocrinol Diabetes Metab 2020; e00176. doi: 10.1002/edm2.176 32904932 PMC7460942

[36] Williamson EJ, Walker AJ, Bhaskaran K, Bacon S, Bates C, Morton CE, et al . Factors associated with COVID-19-related death using OpenSAFELY. Nature 2020; 584: 430–36. doi: 10.1038/s41586-020-2521-4 32640463 PMC7611074

[37] López-Cabrera JD, Orozco-Morales R, Portal-Diaz JA, Lovelle-Enríquez O, Pérez-Díaz M . Current limitations to identify COVID-19 using artificial intelligence with chest X-ray imaging. Health Technol (Berl) 2021; 11: 411–24. doi: 10.1007/s12553-021-00520-2 33585153 PMC7864619

[38] AIX-COVNET, Roberts M, Driggs D, Thorpe M, Gilbey J, Yeung M, et al . Common pitfalls and recommendations for using machine learning to detect and prognosticate for COVID-19 using chest radiographs and CT scans. Nat Mach Intell 2021; 3: 199–217. doi: 10.1038/s42256-021-00307-0

